# Secrets and Misperceptions: The Creation of Self-Fulfilling Illusions

**DOI:** 10.15195/v1.a26

**Published:** 2014-11-03

**Authors:** Sarah K. Cowan

**Affiliations:** New York University

**Keywords:** secrets, communication, social networks, abortion, miscarriage, public opinion

## Abstract

This study examines who hears what secrets, comparing two similar secrets-one that is highly stigmatized and one that is less so. Using a unique survey representative of American adults and intake forms from a medical clinic, I document marked differences in who hears these secrets. People who are sympathetic to the stigmatizing secret are more likely to hear of it than those who may react negatively. This is a consequence of people not just selectively disclosing their own secrets but selectively sharing others’ as well. As a result, people in the same social network will be exposed to and influenced by different information about those they know and hence experience that network differently. When people effectively exist in networks tailored by others not to offend, then the information they hear tends to be that of which they already approve. Were they to hear secrets they disapproved of, then their attitudes might change, but they are less likely to hear those secrets. As such, the patterns of secret hearing contribute to a stasis in public opinion.

In everyday life, we hear about the lives of those we know, and we are, in turn, influenced by what we hear. But sometimes secrets are kept from us. If what we hear and what is kept from us is patterned, then we will be systematically exposed to some influences and not others. This will have implications for all the arenas in which social influence occurs: knowledge, attitudes, norms, and behavior. In this article, I consider how patterns of hearing secrets can affect one outcome: opinions toward that secret. I also discuss how these patterns shape the character and pace of social change more broadly.

We know little about patterns of secret hearing, despite their potential effects on processes of social influence. Perhaps people tend to hear gossip, or stigmatizing secrets, that of which they disapprove. Then they have the opportunity to reevaluate their attitudes, not just to the secret, but also to the person whose secret it is. The contact hypothesis predicts that encountering diverse others can transform people’s attitudes toward minorities and their behavior, usually increasing their tolerance (Williams 1947; Allport 1954; Pettigrew and Tropp 2006). When someone hears the secret that a coworker votes differently than the person does or that the person’s sister has a stigmatized disease, then that contact can effectively occur. Therefore, in the scenario in which we hear stigmatizing secrets, we would anticipate changing attitudes.

It is also possible that secret hearing may not be patterned in this way. Perhaps we are more likely to hear secrets to which we are positively predisposed. Then our social circle would appear to our liking and we would perceive it to be more homophilous than it is. Not-hearing these secrets would produce the false impression that personally objectionable occurrences are rarer among one’s acquaintances than they actually are. Translated into the language of the contact hypothesis, in this scenario, an encounter with diversity would effectively not occur, and we would expect attitudes to remain stable. In either scenario, whether a certain kind of person only hears a certain kind of secret affects public opinion.

A secret is a piece of information deliberately kept from others or specific others for a variety of reasons.^1^ Secret keeping creates an information gap between two people.^2^ In this way, secrets themselves are entirely relational—they are kept from others, and they separate the knowers and the excluded (Simmel 1950; Bok 1989). Furthermore, secrecy is intimately tied to social structure; who keeps what kinds of secrets reflects the culture and power relations within the broader society as well as within the dyad (Simmel 1950; Zerubavel 2006; Nippert-Eng 2010).

The existing empirical work on secrets primarily illuminates firsthand disclosures, demonstrating that people share their own personal secrets with those who will be supportive, avoiding those who will stigmatize (Goffman [1963] 1986; Simmel 1950). This, however, gives us only partial insight into patterns of secret hearing because it misses all the instances in which people share a secret that is not their own to tell—when the officemate shares a piece of juicy news about the boss or the neighbor organizing meal delivery tells the family on the corner about the widow’s terrible diagnosis. In these instances, the secret teller is potentially less likely to know or to care what the predisposition of the secret hearer is than when sharing his or her own secret. To omit these instances of sharing another’s secret would be to omit all these instances of secret hearing, instances that may have a profound effect on the number and characteristics of who hears which secrets, which in turn will affect public opinion.

To capture patterns of hearing secrets, I designed a unique survey that captured how American resident adults hear, hide, and disclose news about two common secrets—abortion and miscarriage. Abortion and miscarriage secrets are a strategic place to begin examining how secrets shape our perceptions of the world and attitudes because they are similar demographic events with markedly different social meanings. Both pregnancy losses are experienced by millions of diverse American women. Having had an abortion, however, is subject to much higher levels of social disapproval and stigma than having had a miscarriage. Though neither abortion nor miscarriage is a joyous event, having had an abortion is understood to speak to the character of the woman more than having had a miscarriage, and women who have had abortions frequently feel stigmatized (Cockrill and Weitz 2010). Given the demographic similarities of the events and the women who experience them, this comparison highlights the effects of a secret’s social meaning on who hears it.

I begin by reviewing the literature on social influence and social networks. This literature tells us how people influence each other and how influence depends on people knowing about each other’s characteristics and experiences. But of course, some of those characteristics and experiences are concealable, and therefore people have the capacity to keep secrets about themselves and others. This will necessarily affect processes of social influence and the attendant outcomes. Given that there are apparently no scholarly studies on patterns of hearing secrets, I next outline the literature on secret disclosure and secret keeping; this is primarily limited to people sharing their own secrets. On the basis of this literature, I formulate a series of hypotheses regarding hearing secrets that I then test. I conclude by discussing the implications of these findings regarding hearing secrets on one particular aggregate outcome: public opinion.

## Social Influence and Social Networks

Network studies and public opinion research find that we influence each other. But to be influenced by another’s characteristics and behaviors, one must know of them. Without that knowledge, social influence is stifled.

Social influence has been shown in a many arenas of social life from innovation adoption (Coleman, Katz, and Menzel 1957) to college academic performance and the likelihood of joining a fraternity (Sacerdote 2001), attitudes toward the homeless (Lee, Farrell, and Link 2004), attitudes toward alternative family forms and practices (Rindfuss et al. 2004), and the likelihood of having a racially diverse set of friends (Emerson, Kimbro, and Yancey 2002), among many others (for examples from a wide variety of settings, see Ryan and Gross [1943], Davis and Greve [1997], Crane [1991], Katz and Lazarsfeld [1955], Huckfeldt and Sprague [1995], and Mutz [2006]).

Awareness of others’ characteristics and behaviors is a necessary condition of all interpersonal influence. To be influenced interpersonally to adopt a new innovation, as an example, one must know that one’s colleague, family, or friends have adopted themselves. This information is often obtained through communication (Rogers 2003), whether the information comes from the focal person directly, or secondhand or thirdhand, and so on, by intermediaries (Friedkin 1983).

Existing formal theories of social influence within social networks rarely consider information flows within the network. Acknowledging communication is a precondition to influence, the scholars simplify the model by assuming either that communication has already occurred or that the probabilities of influential communication are uniform across dyads or topics. They make simplifying assumptions regarding communication and illuminate their central interest—the process of social influence itself, examining the structure of the network and the relationships between people (French 1956; Axelrod 1997; Carley 1991; Friedkin 2006; Marsden and Friedkin 1993; Friedkin and Johnsen 2003; Friedkin and Johnsen 2011). Scholars of diffusion theory take another step by specifically studying a communication network or the individuals in a network who are connected by the flows of information (Rogers 2003).

As this inquiry reveals, even the communication network is a potential network for the transmission of information with regard to a given topic. As an example, all members of a family communicate with each other and are in a communication network together, but there is not complete overlap in the information that will be communicated to each member. When the daughter tells her brother she stole from a neighborhood store, he tells their easygoing father and not their strict mother. Their father is then influenced by that information and treats the daughter differently; the mother never learns and her behavior remains the same. The mother and father have access to different information about the daughter, though they are all in a network, and a communication network, together.

### The Contact Hypothesis

In this article, I focus not on potential communication but on realized communication and how that might differ across dyads and topics; I do so because communication is necessary for the influence process to occur. Take as an example of influence the contact hypothesis which predicts that when individuals come into contact with a stigmatized outgroup, prejudice decreases (Allport 1954; Williams 1947; for reviews, see Jackson 1993; Pettigrew 1998; Brown and Hewstone 2005; for a meta-analysis, see Pettigrew and Tropp 2006). Occasionally, however, contact resulted in increased prejudice (Pettigrew and Tropp 2006).^3^

The contact hypothesis was developed and initially tested regarding intergroup contact between races, ethnicities, and cultural groups but has also shown results for sexual minorities (Herek and Capitanio 1996) and the physically and mentally disabled (Pettigrew and Tropp 2006). It has also shown to be effective for non-ascriptive characteristics such as extra-curricular groups of college students (Biernat and Crandall 1994), people who identify as Republicans or Democrats (Gaertner et al. 1999), and generalist and specialist nurses (Oaker and Brown 1986). Recent scholarship involving a small sample of women finds that when women hear of others’ abortion stories, attitudes shift toward being more positive, particularly among those who previously held negative attitudes (Cockrill 2013).

An optimal condition for contact to change attitudes is if relationships can be formed before the revelation of outgroup status (Pettigrew et al. 2007).^4^ This is possible only with concealable stigmas where trust, affection, and comfort between two people can develop prior to the secret revelation. Nonconcealable stigmas are known at the start of the relationship when rejection is easier than once a relationship is formed.

The revelation of a secret within an already formed relationship has the potential to influence others and change attitudes. When the secret is kept, that potential is unrealized. Individuals then will not know the full truth about those in their social networks,^5^ and social influence within that network is dampened.

Social influence relies on communication, which enables people to learn about each other, particularly about characteristics or experiences that are unobservable. Secret keeping prevents people from hearing about others. Given there is no literature on patterns of hearing secrets, I turn now to the literature on secret disclosure and secret keeping. This literature discusses how people behave with their own secrets; it provides scant information on how people disclose others’ secrets. From this literature I derive hypotheses regarding patterns of hearing secrets.

## Secrets and Secrecy

We can gain some insight into patterns of hearing secrets from the limited empirical work on personal secrets. This literature primarily focuses on people sharing their own secrets, not subsequent disclosures, and it illuminates how the characteristics of the secret both inherent (such as logistical complexity) and social (such as legality, morality and stigma) affect the number of people implicated and the disclosure and secrecy patterns.^6^

### Secret Disclosure

People tell their own secrets because successfully carrying out the secret logistically requires it, because it is unhealthy and challenging to keep secrets, to get emotional support, and to build intimacy.

Some secrets require no or few others to know, such as being HIV positive (Shelley et al. 1995) or being a closeted meat eater in a vegetarian dorm (Kitts 2003). Individuals who are implicated may choose to tell others, but the nature of the secret does not require it. A more logistically complex secret would be obtaining something illegal such as prohibited drugs (Erickson 1981) or an illegal abortion (Lee 1969), or even more so, engaging in a complex crime (Baker and Faulkner 1993; Erickson 1981; Morselli, Giguère and Petit 2007; Bruinsma and Bernasco 2004). These cases require the coordination of multiple actors.

In addition to telling one’s own secrets for logistical reasons, people tell their secrets because keeping them is unhealthy and challenging. Individuals who are harboring a secret display mental fatigue, intrusive thoughts, and negative physiological symptoms (Pennebaker 1989; Pennebaker 1990; Lane and Wegner 1995). They also perceive challenges to be greater and are less likely to help others (Slepian et al. 2012). Disclosing these secrets relieves these effects, however, and so motivates disclosure (Pennebaker 1990). In addition, secret disclosure enables the capacity to get social and emotional support (Emlet 2006).

Secrets also affect relationships; as Simmel described, all social relationships are characterized by the presence and extent of secrecy (Simmel 1950:330). Sharing secrets builds intimacy within adults’ (Richardson 1988) and children’s relationships (Way 2004). Disclosure of personal information increases relationship satisfaction (Finkenauer et al. 2004; Finkenauer and Hazam 2000), feelings of intimacy (Laurenceau, Barrett, and Pietromonaco 1998), and greater emotional involvement (Rubin et al. 1980) and is used within experimental work to create “fast friends” of strangers within the laboratory (Aron et al. 1997; Page-Gould, Mendoza-Denton and Tropp 2008).

There is less empirical work on why people share others’ secrets, but the closely related literature on gossip provides some insights.^7^ Gossip is largely considered trivial or destructive to social life. It does, however, have some social benefits. Gossip is a means of information exchange, allowing one to learn about others in a social circle (Hannerz 1967). Gossiping is fun (Spacks 1982; Ben-Ze’ev 1994). It facilitates social comparison without embarrassment or confrontation (Wert and Salovey 2004). Gossiping enforces group norms and strengthens group bonds (Dunbar 2004) and can situate a gossiper as a person “in the know,” someone who has access to little-known information (Kurland and Pelled 2000). And as an individual, it can provide a similar relief as disclosing one’s own secrets (Feinberg et al. 2012).

Though they are not empirically shown in the gossip literature, we can easily imagine disclosing another’s secret for similar reasons as one might disclose their own, to overcome logistical barriers, to get support, and to build intimacy. Of course, sometimes people misjudge or do not consider the consequences of disclosing a secret, and the disclosure is detrimental to themselves (for an example regarding abortion, see Major et al 1990 [20]), their relationships, or those they care about.

### Secret Keeping

Microsociology of the mid-twentieth century describes individuals withholding their own secrets to maintain social identity and avoid social stigma. Goffman describes concealment as a strategy in stigma management: “because of the great rewards in being considered normal, almost all persons who are in a position to pass will do so on some occasion by intent” (Goffman [1963] 1986:74). As such, stigmas will be revealed selectively depending on the characteristics of the listener (Simmel 1950:314). Empirical work has shown people share their own secrets selectively, at least with regard to illegal abortions (Lee 1969) and being HIV positive (Shelley et al. 1995).

Secrecy can be used to maintain not only reputations but also relationships, which could be damaged by the disclosure of a stigmatizing secret. If the secret is not well received, the revelation may diminish trust because the alter may feel deceived, that ego is not who she had presented herself to be. This would be a violation of trust in identity (Lewis and Weigert 1985; Holzner 1973) or would otherwise disconnect “our idea of a being and the being itself” (Simmel 2004:178). In these situations, withholding the secret can preserve relationships; as an example, withholding secrets within families to avoid stress or pain increases family satisfaction (Vangelisti 1994; Vangelisti and Caughlin 1997).

Confidants may also be compelled to keep others’ secrets for them. Doing so means they avoid appearing as gossips or people who cannot be trusted with sensitive information (Bergmann 1993; Yerkovich 1977; Gilmore 1978), and as such they protect their own reputation. We could easily imagine intermediaries also keeping secrets to preserve relationships—their relationship with the person who told them the secret as well as relationships between the person whose secret it is and others. As an example for the latter case, a sister may learn her brother drank alcohol underage; neither tells their disapproving father to preserve the father–son relationship.

Based on the existing literature, it is evident that individuals will try to share their own secrets selectively to those who will not disapprove of them. There is no clear indication of how intermediaries will share a given secret, whether they will share to those who will approve or will gossip and share to those who disapprove. This can have a marked impact in rates of hearing secrets.

How the information spreads will determine which people have access to what information and influences. It may be that people tend to hear news to which they will react well; or that they tend to hear news which will scandalize them. If secret hearing is patterned, then social networks will be divided by access to information about network members. The boundary of that division will be, at least in part, attitudes toward that information.

Building on insights from the disclosure literature, I have formulated a series of hypotheses that center on the characteristics of the secret, namely, stigma, and the attitudes of potential confidants. This will illuminate whether there are patterns in who hears what secrets and how the patterns of hearing secrets can facilitate social influence and be an agent of social change or can hinder social influence and be an agent of stasis.

## Hypotheses

I propose that fewer people will hear a more stigmatized secret.

*Hypothesis 1:* Among concealable characteristics, the less stigmatized the characteristic is in the wider community, the more people will hear of it and will report knowing someone who has this characteristic.

Considering the literature on stigma, secret disclosure, and gossip, I hypothesize a positive relationship between attitude and hearing secrets.

*Hypothesis 2:* Among concealable and stigmatized characteristics, people who hold positive attitudes toward the characteristic are more likely to hear of it and to report knowing someone who has the characteristic.

If hypothesis 2 is correct, then we should see variation in reporting knowing someone with a stigmatized characteristic by attitude toward that characteristic. This variation, however, could not be a result of communication differences at all but rather merely network segregation. In hypothesis 3, I test that the variation is due, at least in part, to patterns of secret keeping and disclosure.

*Hypothesis 3:* Among concealable characteristics, the more stigmatized the characteristic is, the more likely it is to be disclosed to those who are accepting i.e., the person whose secret it is and subsequent confidants will reveal the concealable information to persons least likely to “punish” the person whose secret it is for that revelation.

While I have made a case for these hypotheses, the data we have as of yet only illuminate firsthand disclosures. People can hear of a secret from the person whose secret it is or from someone else. Without data on these latter disclosures, we cannot test any communication mechanism to explain patterns of hearing secrets. I have proposed that secrets will be more likely told to those who are supportive than those who will react negatively. I outlined previously how if secrets are revealed in this manner, then this will heighten the experience of homophilous networks, above and beyond the network’s objective homophily. This exacerbation would stifle attitude change. If, however, secrets are not disclosed in this manner, if, for example, people share secrets as if they are juicy gossip disclosing to those who will react negatively, then a heightened experienced homophily will not occur. Then patterns of secret keeping and selective disclosure may contribute to social change. To empirically consider this requires capturing patterns of hearing and disclosing secrets whether the disclosure is first-hand or made by confidants. I present one case, comparing abortion and miscarriage secrets.

## Empirical Strategy: Comparing Abortion and Miscarriage Secrets

To test the hypotheses, I compare miscarriage and abortion secrets in the United States. These secrets are a strategic place to test my hypotheses because of their demographic similarities and social differences. Miscarriages and abortions are both common events that end pregnancies, are usually concealable, and are experienced by millions of diverse women (many of whom experience both events). These pregnancy losses differ, importantly, in regard to associated stigma. Therefore, differences I find in the communication of these events can likely be attributed to differences in the social meaning of the event rather than differences in the women experiencing them.

One in three American women will have an abortion in her lifetime at current rates (Jones and Kavanaugh 2011), and an estimated 1.2 million abortions were performed in the United States in 2008 (Jones et al 2008). Nearly 20 percent of recognized pregnancies end in abortion (author’s calculations from Ventura et al. 2012).

Women who have abortions have many similar characteristics as women of childbearing age generally. The patient population is comparable to women of childbearing age with regard to religion, motherhood, partner status, and education. For example, in 2008, just over a quarter of abortion patients were Catholic, as were just over a quarter of American women aged 15–44. Sixty percent of women who have abortions are mothers; 56 percent of women aged 15–44 are mothers. Almost half of women who have abortions are married or cohabiting; just over half of women of reproductive age are married or cohabiting. Fifteen percent of women who have had an abortion attend a religious service at least once a week, as do 24 percent of women aged 15–44. The educational attainment of women who have had abortions matches almost precisely the educational attainment of American women aged 15–44 as a whole. There are, of course, some differences between the demographics of the women of childbearing age in the United States and that of abortion patients. Black and Hispanic women are overrepresented among abortion patients, as are women aged 20–30. Women whose family incomes are less than the federal poverty limit are overrepresented, and women whose family incomes are more than 200 percent of the federal poverty limit are underrepresented (Jones, Finer, and Singh 2010). Nevertheless, the one in three women who will have an abortion by the time she reaches 45 are drawn from all subpopulations of American women.

Miscarriage is less common than abortion but is still highly prevalent. Of recognized pregnancies, approximately 13 percent end in miscarriage (Stirrat 1990; Goldhaber and Fireman 1991; Rai and Regan 2006). Given available data, it is impossible to precisely determine how many women have had miscarriages to compare it to abortions (Weinberg et al. 1992; Wilcox and Horney 1984). It is certain fewer recognized pregnancies end in miscarriage than abortion and likely that there are fewer women who have experienced miscarriages than abortions.

Women who have had any miscarriage are representative of the population generally as the majority of first miscarriages are due to random fetal chromosomal abnormalities. The risk of these abnormalities increases with maternal age but is understood to be mostly a random event (Wilcox et al. 1988; Rai and Regan 2006). In the multivariate analyses that follow, I exploit miscarriage as a near-random event in the comparison to abortion.

Despite its widespread prevalence, stigma concerning abortion is dramatic and more severe than stigma concerning miscarriage. Women are disinclined to disclose their abortion histories (Major and Gramzow 1999) and perceive strong social disapproval in nearly every context (Cockrill and Weitz 2010). The most compelling evidence that miscarriage is less stigmatized than abortion is that women frequently report their abortions as miscarriages to doctors and survey researchers, among others (Jones and Kost 2007; Erviti, Castro, and Collado 2004). Furthermore, abortion is seen as a choice, whereas miscarriage is not. As such, women who terminate pregnancies are much more likely to have feelings of guilt and shame after the procedure than women who miscarried (Keefe-Cooperman 2005; Broen et al. 2004, 2005). To be sure, some women who miscarry feel a sense of stigma, but it is usually due to interpreting miscarriage as a sign of infertility (Miall 1985) rather than the stigma of abortion, which can be seen as a sign of the woman’s promiscuousness, irresponsibility, and immoral character.

This inquiry addresses who hears abortion and miscarriage secrets. Both miscarriages and abortions usually happen early in the pregnancy and hence are concealable (Henshaw and Kost 2008). As a near-random event with little stigma and little selective disclosure, Americans of all subpopulations have about even rates of hearing about another’s miscarriage (see Appendix B in the online supplement). It is thus an ideal benchmark for comparing rates of hearing an abortion secret. In addition, the inclusion of miscarriage allows me to account for otherwise unobservable characteristics that may affect whether individuals hear an abortion secret such how common pregnancies are among those they know and how often respondents hear of those pregnancies.

If secrets were told without care to listeners’ attitudes, then differences in rates of hearing secrets would merely reflect differences in network composition. I address this concern, at least partially, through choosing test secrets that are common and that diverse women have experienced. The analytical strategy I employ further accounts for network segregation on the basis of major demographic characteristics such as age, gender, political party, and race, among others. This is discussed more in what follows. Lastly, I include analyses on the patterns of disclosure and the motivations to share and keep secrets to test whether there are differences in communication that could exacerbate any underlying network segregation.

## Data

### Survey Sample

Data to test the hypotheses come from a nationally representative survey of American adults I designed and administered for this study; it is called the American Miscarriage and Abortion Communication Survey (AMACS) and was administered in the winter of 2012. The sample is more than 1,600 adults in the United States. The survey was conducted over the Internet as individuals report higher rates of sensitive behaviors than in other methods (Schroeder, Carey, and Vanable 2003).

The survey was implemented by the firm GfK, known as Knowledge Networks (KN) at the time of the survey. Knowledge Networks uses a prerecruited probability-based web panel (Callegaro and Disogra 2008). Respondents are recruited into a panel of 50,000 through random-digit dialing and address-based sampling methods. By joining the panel, respondents agreed to participate periodically in online surveys and were provided Internet access and equipment if they did not already have it. As such, this Internet survey includes individuals who otherwise would not have participated in Internet surveys due to lack of access. Respondents in the panel are asked to fill out an initial profile of basic demographic information. This study had a 64.9 percent profile completion rate. Three thousand panel members were invited to specifically take the AMACS survey, of which 1,640 completed the survey, a completion rate of 54.7 percent.

Knowledge Networks’s samples closely match those of traditional RDD surveys and are representative of the United States as a whole (see Chang and Krosnick [2009] for KN’s RDD samples; see DiSogra, Dennis, and Fahimi [2010] on ABS). KN samples are used extensively in academic and government research, including the American National Election Survey and the Time-Sharing Experiments for the Social Sciences (TESS). This data collection was its own survey, not one bundled with others or a part of the TESS experiments.

In addition to the benefit of higher disclosure rates, administering the survey over the computer avoids interviewer fatigue. Interviewers can fatigue of the name generator questions as I employ (and as discussed later) thus affecting data quality (Paik and Sanchagrin 2013).

Though abortion and miscarriage are events only women experience, this inquiry hinges on Americans’ rates of hearing about abortion and miscarriages. Both men and women can hear about these events, and so the sample for this survey is representative of American resident adults generally.

The data are weighted to adjust for known sources of deviation from an equal probability of selection design. To reduce the effects of non-coverage or nonresponse bias, a poststratification adjustment is applied using demographic distributions from the most recent data from the Current Population Survey (CPS) for gender, age, race and ethnicity, education, census region, and whether the respondent lives in a city. The results are also weighted with regard to Internet access, data on which are collected at time of recruitment. All reported results are weighted.

### Survey Design

The AMACS survey captures how abortion and miscarriage secrets spread by asking American-resident adults four modules on their knowledge of others’ experiences of miscarriage and abortion and about their own experiences of miscarriage and abortion (men are asked about their partner’s experiences). I provide a brief description of the survey here; Appendix A in the online supplement has more detailed information. Each of the four modules is structured to allow comparisons across modules. Respondents are randomly assigned to answer questions about others’ abortions or miscarriages first so as to avoid any ordering effects. Having finished a module on one, they then answer the other. For parsimony’s sake, I will only describe the modules on miscarriage; the abortion modules are precisely the same, except the questions ask about abortion.

Respondents reveal whether they know a woman who has had a miscarriage. If they do, they are then asked a series of questions regarding the most recent miscarriage they heard of, whether they knew about the pregnancy prior to the miscarriage, and their relationship to the woman, among other questions. After respondents provide details on the most recent event, they provide information on whom they told about the miscarriage through a series of name generator questions for their immediate family, close friends, and anyone else. They are also asked an open-ended question regarding the reasons they told specific people.

Having provided details on whom respondents told, respondents are asked questions about from whom they deliberately kept the pregnancy loss a secret. Respondents are asked, “Is there anyone you usually talk with about personal matters but you deliberately did not tell them about this miscarriage?” In exactly the same manner as in the module where respondents outline to whom they told the secret, they outline from whom they withheld the information and why.

After having answered questions about their knowledge of other people’s abortions and miscarriages, respondents answer two modules on their own experience with these events. They are randomly assigned to answer questions about miscarriage or abortion first. Again, these modules are structured so as to make comparisons with the other modules in the survey. Respondents answer questions about the most recent event, whom they told and why, and from whom they withheld the information.

There is a fifth module with standard demographic, network, and attitude questions. In it, respondents provide their attitude toward abortion; they are asked, “Which of these comes closest to your view?” There are then four possible answers provided: abortion should be generally available to those who want it; abortion should be available but under stricter limits than it is now; abortion should be against the law except in cases of rape, incest, and to save a woman’s life; abortion should not be permitted at all.^8^

It is certainly the case that one’s attitude toward abortion generally may not represent one’s attitudes toward a particular abortion about which one may or may not hear. The generalized attitude is the public information about which egos determine whether to share the secret of a particular abortion, and so it is the attitude included in these analyses.

Respondents also answer two questions aimed at representing how gregarious they are, from which I created a gregariousness scale.^9^ Respondents are randomly assigned to take the demographics module before or after the four modules on abortion and miscarriage.

Underreporting of abortion is well documented (Jones and Kost 2007). As is seen in what follows, the reporting in AMACS of respondents’ own abortion indicates that this survey also suffers from underreporting. Therefore these data should not be taken as an indication of the true prevalence of this event. Those who report having experienced a pregnancy loss within a survey are a select group of those who have experienced pregnancy loss. Nonetheless, the data provide insight into how these secrets diffuse. I anticipate the rates of telling and numbers of others told are overestimates given I have data only on those willing to report an abortion within a survey. As such, the differences reported between abortion and miscarriage rates will be conservative.

### Clinic Data

While the AMACS data are the primary data used in these analyses, I also draw on a unique data set collected at an urban abortion clinic that serves patients from the region. These data provide insight into the beginnings of the disclosure of this secret. They are counseling intake forms for more than 5,000 women who presented for an abortion in 2008. The forms were a part of routine care at a privately owned, dedicated abortion clinic in a state that does not mandate parental involvement for minors seeking abortion services. Crucial for this study, the women indicated who knows that they are at the clinic and whether the confidants supported their decision (more information on these data can be found in Foster et al. [2012]).

Demographic information on the patients was obtained from other intake forms. Those data reveal the abortion patients in the clinic are similar to abortion patients nationally though less likely to be of Hispanic origin. This unique data set sidesteps the underreporting that is a well-documented pitfall of surveying women about their abortions (Jones and Kost 2007). It avoids this problem in two ways: first, the women have already revealed they are having an abortion by appearing at the clinic, and second, the data collection is not a part of research but rather routine care. The clinic data will be used along with the AMACS survey data to test hypothesis 3.

## Results

The AMACS survey data show that miscarriage and abortion secrets are heard at different rates by different Americans. Three-quarters of Americans say they know someone who had a miscarriage; half report knowing someone who had an abortion. Given that abortion is more common than miscarriage (as discussed earlier, utilizing nonsurvey data), this is a striking indication that abortion secrets have not been communicated as often as miscarriage secrets. This evidence supports hypothesis 1.

All demographic subgroups report higher rates of knowing a woman who has had a miscarriage than an abortion. While there is some small variance in differences in knowledge across subgroups, they are as expected: women hear more miscarriage and abortion secrets than men; older Americans have heard more secrets than younger Americans, except for the oldest group. Appendix B provides all the bivariate statistics.

That more Americans report knowing someone who has had a miscarriage than an abortion is a result of both secret keeping and secret disclosure. Miscarriage secrets are told to more people than abortion secrets and are kept from fewer people than abortion secrets, as can be seen in Table 1. Seventy-seven percent of women and their partners who experienced a miscarriage go on to tell someone else; for each miscarriage, they tell, on average, 2.63 people. Sixty-six percent go on to share their abortion secrets to an average of 1.24 people. As such, for each miscarriage, two people are told initially, and for each abortion less than one person is told initially. All these differences are statistically significant.

Intermediaries share miscarriage secrets more frequently and to more people than abortion secrets. Thirty-one percent of miscarriage secrets are shared, whereas only 16 percent of abortion secrets are (*p* < 0.001). When intermediaries did share another person’s miscarriage secret, they told 2.73 people on average, and when people shared another person’s abortion secret, they told 2.22 people on average (*p* < 0.1). As such, for each person told about another’s miscarriage, 0.85 people were subsequently told; and for each person told about another’s abortion, 0.35 people were subsequently told (*p* < 0.001).

Not only were abortion secrets told to fewer people than miscarriage secrets, they were kept from more people. Thirty-one percent of individuals who experienced an abortion specifically avoided telling someone with whom they usually speak about personal matters. If they avoid anyone, they avoid 2.6 people on average. Individuals who experienced a miscarriage avoided a similar number of people on average, but many fewer of them keep the secret at all, only 7 percent. Therefore, for each abortion, 0.8 people are avoided, and for each miscarriage, 0.2 people are avoided at this initial stage (*p* < 0.01). Regarding others’ pregnancy loss, 25 percent avoid disclosing an abortion and 13 percent avoid disclosing a miscarriage (*p* < 0.001). More people are avoided for a miscarriage, but because fewer people avoid anyone at all, overall 0.47 people are avoided when disclosing a miscarriage and 0.74 when disclosing an abortion (*p* < 0.001).

The process of secret keeping and selective disclosure begins even before the pregnancy loss; women are more restrictive in whom they tell about pregnancies that will end in abortion than in miscarriage. Sixty percent of respondents who report knowing someone who had a miscarriage say they had already known about the pregnancy, whereas only 24 percent said they knew about the pregnancy in advance of hearing about the abortion.^10^

Though abortion is a more common occurrence than miscarriage in the United States, more people reported knowing someone who has had a miscarriage than an abortion. Table 1 illustrates how this difference arises—not only are fewer people informed about an abortion, the abortion secret is kept from more people than are miscarriage secrets.

Within families, abortions are kept secret much more often than miscarriages, as seen in Table 2. Nearly all Americans who experience a miscarriage tell a member of their family, whereas less than three-quarters of individuals who experience an abortion do (*p* < 0.001). Of people who report knowing about someone else’s miscarriage, 19 percent are the immediate family of the woman; for abortion, this is only 11 percent (*p* < 0.001). In contrast, it is more common that acquaintances know about an abortion (33 percent) than a miscarriage (27 percent; *p* < 0.01). It is also more common for a boyfriend or girl-friend to know about an abortion (8 percent) than a miscarriage (4 percent; *p* < 0.001). It is important to note that given the structure of the survey, this boyfriend was not the man involved in the pregnancy but more likely a boyfriend in a relationship that started after the event. The rates of friends and others knowing are similar for miscarriage and abortion.

Americans similarly keep their own abortions secret more frequently than they keep their own miscarriage secrets. Individuals who experience an abortion are more likely to avoid telling close friends or individuals of another relationship than individuals discussing their own miscarriages. These differences are large in magnitude but not statistically significant due to sample size. Eighty percent of people keep their abortions or miscarriages secret from a member of their immediate family.

When discussing others’ experiences, respondents are also more likely keep abortion secrets from their immediate family than miscarriage secrets. Often these individuals are keeping a secret about one family member’s pregnancy loss from another family member, as in a brother who conceals her sister’s pregnancy loss from their parents. These secrets are also kept from confidant’s family members, as an example, a wife who will not share with her husband the news of her friend’s miscarriage. DiPrete et al. (2011) show families are an integrative arena in the United States and they explain this in part because of the difficulties in keeping secrets within families. Pregnancy losses, especially abortions, however, are often kept secret within families.

Though abortion is more common, significantly more people will learn that someone they know has had a miscarriage than an abortion. This stands in support of hypothesis 1, that among concealable characteristics, more people will learn of another’s less stigmatized characteristic than a more stigmatized one. This difference is due to the patterns of disclosing and withholding secrets which favor the spread of miscarriage secrets compared to abortion secrets. The couple experiencing pregnancy loss, and subsequent confidants share miscarriage secrets more frequently and to more people than abortion secrets. This is a likely but not an inevitable relationship between stigma and the disclosing and withholding of information. Analyses of other secrets will be necessary to fully test hypothesis 1.

**Figure 1:**
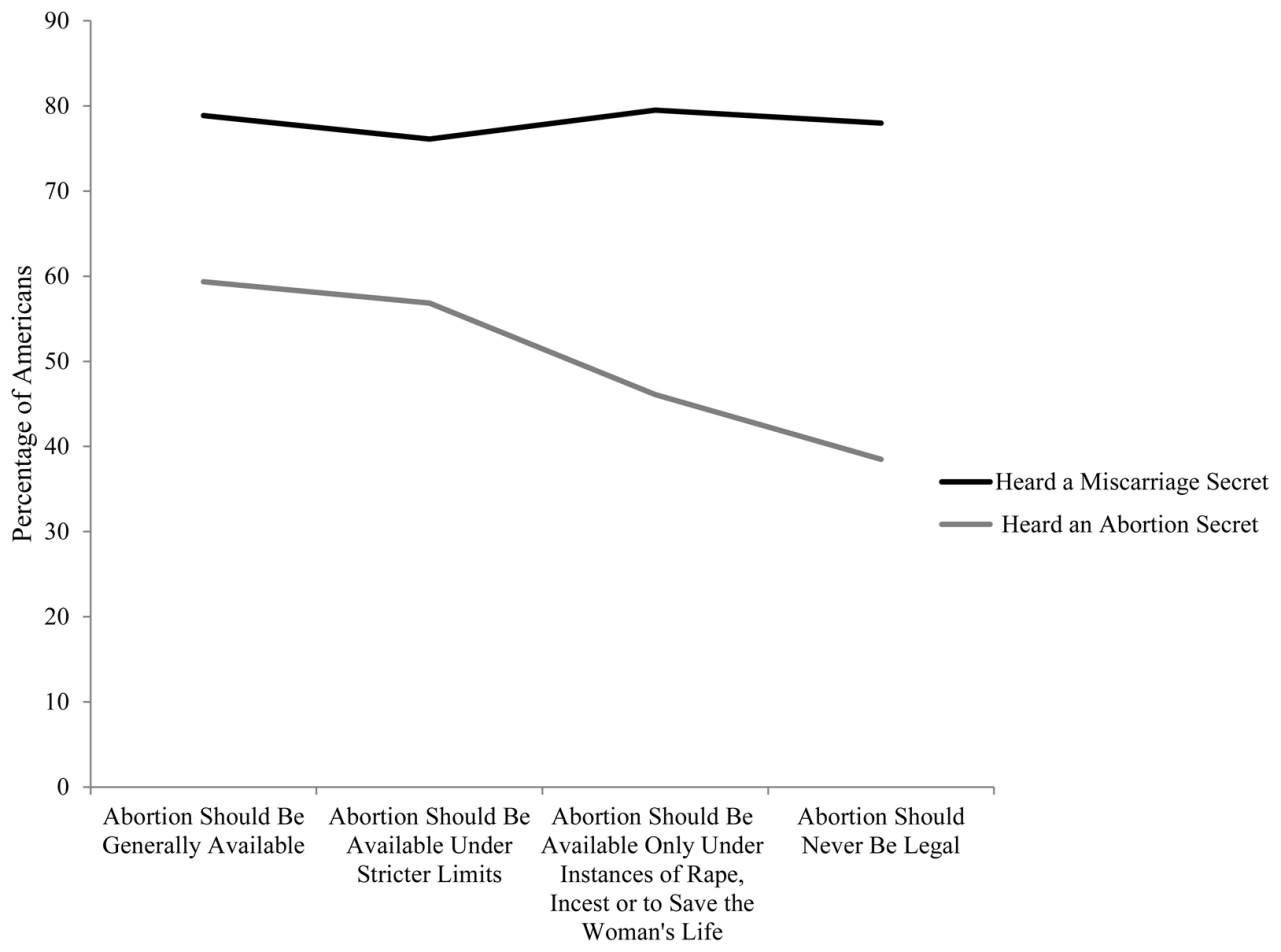
Percentage of Americans who have heard about someone else’s abortion and miscarriage by own attitude toward abortion legality. AMACS 2012. Note the full descriptive statistics of the rates by demographics are found in Appendix B.

Hypothesis 2 states that respondents who have liberal views toward abortion are more likely to hear abortion secrets regarding the women they know than people who have conservative views. As we can see in Figure 1, pro-choice Americans are more likely to hear abortion secrets than anti-abortion Americans. One’s attitude has no bearing on hearing miscarriage secrets. Almost 60 percent of respondents who believe abortion should be generally available (40 percent of the sample) report knowing a woman who has had an abortion. Fewer than 40 percent who think abortion should never be legal (13 percent of the sample) report knowing a woman who has had an abortion (*p* < 0.001). Regardless of respondents’ attitudes toward abortion, about 80 percent know someone who has had a miscarriage.

Table 3 reports regression results which demonstrate this relationship between attitude and contact holds within a multivariate analysis. The four models presented in Table 3 all demonstrate that Americans who hold conservative views on abortion are much less likely to hear of someone’s abortion than their more liberal counterparts. These are logistic regression models predicting whether a respondent reports knowing someone who has had an abortion. Reported in the table are odds-ratios; in what follows, when discussing the relationship between abortion attitude and having heard an abortion secret, I report relative risk ratios for ease of interpretation. Model fit was diagnosed using Hosmer–Lemeshow’s *F*-adjusted mean residual test for logistic regression using sample survey data (Archer and Lemeshow 2006). All of the models control for the randomization of modules within the survey; this does not have a substantive effect on the results and are not reported.^11^ Model 1 is the simple bivariate analysis using only the independent variable of interest—attitude toward legal abortion.

The second model additionally controls for the number of miscarriage secrets the respondent has heard which captures how likely the respondent is to hear secrets, particularly pregnancy-related secrets. In the presence of this control, there is still a significant gradient in hearing abortion secrets by attitude toward abortion. The third model leaves out the miscarriage secret control but includes a series of demographic variables.

The full model, model 4, predicts hearing an abortion secret while controlling for the full set of demographic variables and the unobservable attributes which are captured by respondent’s knowledge of others’ miscarriages. It remains the case that Americans who hold restrictive views on abortion are much less likely to hear of others’ abortions than Americans who hold liberal views on abortion. Americans who believe abortion should be legal under no circumstance are 21 percent less likely to have heard an abortion secret than those who believe abortion should be generally available (*p* < 0.01). Those who believe abortion should be legal only in the cases of rape, incest or to save the life of the pregnant woman are 12 percent less likely (*p* < 0.05). Holding these attitudes is one of the largest predictors of reporting knowing someone who has had an abortion.

While one’s attitude toward abortion is a significant predictor of hearing abortion secrets, a few control variables are significant predictors as well (not shown). Older Americans are more likely than younger Americans to hear these secrets (*p*-values range from *p* < 0.05 to *p* < 0.001). Men are less likely than women (*p* < 0.001). Respondents who identify their racial and ethnic status as other, non-Hispanic are less likely to hear of an abortion among the women they know than white, non-Hispanic respondents (*p* < 0.05). Individuals who have attended some college are more likely than Americans who did not graduate from high school to report knowing someone who has had an abortion (*p* < 0.05). Individuals in the second-highest income bracket are more likely to report knowing someone who has had an abortion compared to the lowest income bracket (*p* < 0.01). Respondents who identify as Evangelical are more likely to report knowing someone who has had an abortion (*p* < 0.01) than respondents are not Evangelical. As expected, more gregarious respondents are more likely to hear abortion secrets (*p* < 0.05).

This all stands in support of hypothesis 2, that pro-choice Americans are more likely to hear abortion secrets than anti-abortion Americans. This pattern could result from a number of mechanisms, including assortative mixing. Perhaps more pro-choice Americans know a woman who has had an abortion history; perhaps networks are segregated with respect to both abortion prevalence and attitude. Given the regression analysis controls for demographic and socioeconomic covariates, we can consider two people who are precisely the same with regard to their background characteristics but differ with regard to their opinion on abortion. Even when there exists network segregation based on race, age, and other background characteristics, two people who are identical with regard to the demographic characteristics included in the analysis above should be equally likely to have a women with an abortion history in their network. Controlling for these demographic characteristics, we see a substantial difference in the likelihood of hearing an abortion secret by one’s attitude toward abortion. The one of the two people who is most vehemently opposed to abortion is significantly less likely to hear of another’s abortion than the one who is pro-choice. Were this pattern the result of assortative mixing, it would necessarily be the result of other factors related to both abortion opinion and the likelihood to know a woman with an abortion history that have not been used in this regression model. Therefore the fact that people who hold anti-abortion attitudes are less likely to hear an abortion secret is suggestive of secret keeping and selective disclosure on the basis of opinion, and we now turn to hypothesis 3 to examine this further.

To test hypothesis 3, I examine two data sources. First, I consider the clinic data, examining whether the confidants of the abortion patients were supportive. Then, I turn to the qualitative responses from the AMACS survey, where respondents explained why they disclosed or kept secret their own or others’ pregnancy losses. In both these data sources, I find that women who have had abortions and subsequent confidants tend to keep abortion secrets from those who will stigmatize and this helps explain why Americans who are pro-choice more frequently hear abortion secrets than Americans who are anti-abortion.

The clinic’s patients overwhelmingly disclose to people who are supportive. Over 80 percent of the confidants are supportive; this figure rises to over 90 percent when I exclude male partners who are the least supportive group and arguably the group women may feel most obliged to tell. This far exceeds the 48 percent of Americans who are pro-choice. This suggests women are sharing their decision to get an abortion selectively; they seek out those they anticipate will support them and avoid those who will punish them. This supports hypothesis 3 that disclosure will be channeled along lines of preexisting acceptance.

While the clinic data gave insight into the patterns when individuals share their own secrets, the AMACS data illuminates patterns of revealing someone else’s secrets as well. Respondents spread the news of an abortion or miscarriage selectively, telling some and avoiding others; they explained why. When talking about their own pregnancy losses or someone else’s, Americans informed others for largely the same reasons. Abortion and miscarriage secrets were kept, however, for quite different reasons.

Americans most commonly disclose a miscarriage or an abortion—either one’s own or another’s—to receive support or because they have an intimate relationship such as being family or close friends.^12^ As an example, in explaining why she told a friend about another woman’s miscarriage, a respondent writes, “She and the woman are close friends. She is a seminary student with an emphasis on hospital chaplaincy—she could minister to her friend. We could do something together to make the young woman feel better—we shared a quiet meal and listened/chatted about whatever she was feeling.” Another explains why she told her sister about her miscarriage, “She is my sister. She did not know that I was pregnant to begin with, but the day after I had my miscarriage, I broke down and told her. As I sat there and cried, she just held me in her arms and just listened. She was the most supportive person that day.” A mother wrote how she handled her daughter’s friend’s abortion; “This girl was our daughter’s friend. We love her. We told another friend because she needed to know so she could help.”

Abortion and miscarriage secrets are also told as a means of explanation. As an example, if the confidant already knew about the pregnancy, they would be notified about its loss so as to explain why the woman was no longer pregnant. Additionally, respondents cite having to explain an absence from work or a family event as reasons for disclosing the pregnancy loss.

Differences arise in why miscarriages and abortions are kept secret. This is illustrated in Table 4, which reports a quantification of the AMACS data, where respondents give qualitative answers to why they did or did not share news of a pregnancy loss. Both women who have experienced a pregnancy loss and subsequent confidants keep these secrets to preserve privacy. Abortion secrets are also withheld to avoid stigma whereas miscarriages secrets are not. One’s attitude toward abortion causally determines whether one is knowingly exposed to someone who has had one. In this regard, the contrast between miscarriage and abortion is stark.

Privacy is the most common reason for keeping both miscarriage and abortion secrets. As an example, one respondent wrote, “Judith knows the person who miscarried and would be told by the person herself if she wanted to share information… if I asked, I may reveal private information.” Another writes about not revealing an abortion, “The affected person’s past decisions are nobody else’s business; if she wanted other people to know she is the one to tell them not me.” Privacy is about as common a reason for keeping miscarriage and abortion secrets. Some couples reveal their experience to confidants but specifically ask for their secrecy; secrets are kept from 29 percent of potential confidants for this reason for abortion while only 13 percent for miscarriage (*p* < 0.01). This difference is suggestive of the stigma associated with abortion but not miscarriage.

Explicitly avoiding stigma is a much more common reason for keeping abortion secrets compared to miscarriage secrets. Many secrets are kept—by individuals experiencing the abortion and subsequent confidants—specifically to avoid judgment. Of the people individuals avoided telling about their own abortion, 36 percent were due, at least in part, to avoiding stigma. For miscarriage, that is less than 3 percent (*p* < 0.001). One respondent writes about her abortion, “My dad would have been upset with me. He would have judged me. I really love my dad and have a close relationship with him. I did not want him to feel disappointed.” Another writes about not telling her mother about her abortion: “She would have been mean and not understanding about it. She would have tried to make me feel horrible. We did not have a relationship of unconditional love; everything with her had a condition… her condition. It was my own problem, I asked her for no help or understanding.”

People similarly withhold other people’s abortion secrets from those who may reject or punish the woman who had the abortion. Avoiding stigma was given as a reason to keep an abortion secret 13 percent of the time and just 2 percent of the time for miscarriage secrets (*p* < 0.001). One woman explains why for years she has kept her close friend’s abortion a secret from her father: “My dad is a very judgmental person and a Mormon.” Another woman writes about keeping her friend’s abortion a secret from her mother: “Too judgmental a person to tell something that personal to. She would never let it go and it was simply not her business nor could she be open-minded enough to understand it.” A man explains why he kept an immediate family member’s abortion a secret from a friend: “This is a very private and personal matter which would affect how they view this person.” Some individuals express a lack of stigma around miscarriage as a justification for sharing the information. As one man writes about sharing the news of a friend’s miscarriage, “It’s acceptable to talk about miscarriage; a person doesn’t look like a killer.”

The clinic data and the qualitative AMACS data indicate that individuals carefully manage both their own and someone else’s abortion and miscarriage secrets. They share secrets with those who will be supportive and, in the case of abortion, withhold from who might react negatively. This confirms hypothesis 3, the more stigmatized piece of information has a channeled disclosure; the secret travels to those who will be accepting, avoiding those who will not.

Given that pregnancy loss is concealable, individuals have the opportunity to manipulate who knows this information and who does not. Anti-abortion Americans are less likely than pro-choice Americans to hear an abortion secret, even controlling for demographic, socioeconomic, and discussion factors. These different propensities to hear an abortion secret exist within the same network, even within the same family, and secrets are kept and told accordingly. Each individual then experiences a somewhat different network composition even within the same objective social network, and that experience is determined by preexisting attitudes.

## Discussion

Though abortion is a more common event in the United States than miscarriage, this article shows that more Americans hear of women who have had miscarriages than they hear of women who have had abortions. This is a result of both the patterns of secret telling and keeping: more Americans tell miscarriage secrets to more people than abortion secrets, and more Americans keep abortion secrets from more people than miscarriage secrets.

In the introduction, I described two scenarios: one in which people tend to hear secrets they previously approved, and this pattern would contribute to a stasis in public opinion and a second scenario in which people hear secrets they previously condemned and this scenario would inspire social influence and facilitate social change. The data analyzed here illustrate the first scenario. They show a strong trend whereby individuals who hold restrictive views toward abortion are less likely than their liberal peers to report knowing someone who has had one. People tend to hear those secrets about which they already approve and are less likely to hear secrets about which they disapprove. Secret keeping and selective disclosure intensify this experience of homophily above and beyond any objective network segregation.

As outlined earlier, if people are more likely to hear about that which they approve, then processes of social influence will be dampened, and we would anticipate stable attitudes. That is precisely what we see with regard to abortion attitudes. Americans by and large do not change their attitudes on abortion over the course of their lives. Eighty-five percent of the AMACS respondents report their abortion opinion has not changed in the past few years. Panel studies have also found stability in individuals’ abortion attitudes over time (Norrander and Wilcox 1999; Hout and Hastings 2012).

Secret keeping that prevents contact between those who are opposed to abortion and women who have had them helps maintain this attitude stability. The few respondents in this study who did change their abortion opinion cite personal contact. One woman in the AMACS sample explained that she had become more opposed to abortion rights in the last few years because she knew of “too many instances where abortion was used instead of contraception.” Another woman explained her increased support of abortion rights by referencing how seriously individuals approach the decision: “I am dear friends with many who have had abortions… I understand why many choose abortion.”

This is confirmed by the case of rapid attitude change in the United States with regard to sexual minority rights. This change toward more liberal attitudes is due, some scholars argue, to the increasing personal contact between Americans in general and sexual minorities (Lewis 2011).

As we see from the AMACS respondents and the case of sexual minorities, individuals’ attitudes can be influenced and changed by personal information, but personal information about abortion is being carefully managed. The literature on social influence, in particular the contact hypothesis, indicates that were people who are opposed to abortion to hear that women they know have had abortions, then their opinions would likely change. They may become more liberal toward abortion or more conservative, but they would be influenced by this news. But when there is silence rather than discussion, individuals cannot influence each other, and attitudes remain stable.

I am introducing a new route to the experience of homophilous networks, secret keeping. This homophily is not just a result of individuals choosing to be with those who agree with them or behaving as they like but also because the people they are with imply that they do—they hide what will be met with disapproval. With this mechanism, two people within the same network can experience it differently depending on which secrets they hear.

Differences in the rates of hearing any secret will be due to a combination of network segregation and secret keeping, but we have reason to believe that in the case of abortion and miscarriage secrets, secret keeping and selective disclosure are the primary mechanisms to explain these differences. The abortion patient population is large and varied, and millions of diverse Americans hold different attitudes toward abortion. Furthermore, the analyses presented here included a wide range of demographic and socioeconomic characteristics that are often the source of network segregation. Given this, it is unlikely there is an unobserved characteristic that segregates networks with regard to both abortion history and attitudes toward abortion.

In theory, the differences in rates of hearing secrets could be a result of interpersonal influence—that Americans come into contact with women who have had abortions, are influenced by their stories, and become more liberal. Then they would appear in the survey as pro-choice and knowing someone who has had an abortion but the change in attitude would have occurred prior to the survey. As discussed, Americans rarely change their attitudes on abortion, so this indicates that interpersonal influence is only marginally at play here. More likely, secret keeping and selective disclosure prevent the contact necessary for social influence.

Selective disclosure and secret keeping do more than help explain these patterns in contact by attitude; they are mechanisms for thwarting social influence by preventing the awareness that is a precondition of influence. Social influence is predicated on individuals having information about those around them; when that information is absent, social influence cannot proceed. The stability of Americans’ attitudes toward abortion in the United States provides one example.

## Conclusion

This inquiry has examined secret sharing and withholding on a large scale with data representative of Americans nationally. I find secrets of a stigmatizing nature are told to fewer people and kept from more people than secrets that are less stigmatizing. Furthermore, stigmatizing secrets are channeled away from individuals with preexisting negative attitudes. A person who views a secret favorably will more likely hear of these secrets and believe those secrets more common among acquaintances, friends, and family than a person who views a secret negatively and does not hear these secrets. This is the case even if they are in the same social network. Scholarship has already shown that social networks shape attitudes; I show that through secret keeping and sharing, attitudes shape individual experiences of social networks as well.

The combination of secret keeping and selective disclosure enables individuals to perceive their social networks to be different than they actually are. When secrets are told to the approving and kept from the disapproving, networks appear as the focal person or ego prefers, and this person effectively lives in a homophilous network. The existence of homophilous networks is usually explained three ways: first, individuals become similar to their social networks through processes of interpersonal influence; second, individuals select social networks of similar others; and third, structural factors result in similar individuals being in the same social networks. Here I empirically demonstrate that selective disclosure and secret keeping provide a fourth route to ego experiencing a homophilous network—ego can exist in a network where people do not hold a person’s attitudes or behave as the person would like but the person mistakenly perceives that they do. Homophilous networks thwart social influence and contribute to stasis, and we see that with regard to public opinion on abortion.

Secret keeping has further implications for the dialectical process of perceiving and creating the social world described by social constructionists as the process that produces the “social stock of knowledge” (Berger and Luckmann 1967) or “socially derived knowledge” (Schütz 1982). I have shown that individuals can exist within the same objective world but perceive that world differently. As such, the creation of social worlds through conversation is distorted. This bias and distortion are along lines of preexisting attitudes and are biased toward individuals experiencing a network amenable to them.

The little sociological research on secrets examines how people behave with regard to their own secrets. I shift the focus to hearing secrets, and this reveals that selective disclosure of secrets permits a self-fulfilling illusion; those who are opposed to a given secret are less likely to hear of it even if it exists in their social vicinity. They then do not have to face the truth about those they know and confront their own beliefs about the secret and those implicated in it. Had they, they might have engaged in a process of social influence and changed their beliefs, but when secrets are kept from them, they do not have that opportunity. Absent that opportunity, stasis will likely prevail.

## Figures and Tables

**Table 1:** Frequency and Magnitude of Secret Telling and Secret Keeping for Own and Others’ Miscarriages and Abortions, AMACS 2012

	Respondent or Partner Has Had:		Diff.	Respondent Has Heard of Someone Else’s:		Diff.
	Miscarriage	Abortion		Miscarriage	Abortion	
Secret telling						
Respondent disclosed secret (%)	77.31	66.00	+	31.14	15.85	+
If disclosed, mean number of people told	2.63	1.24	†	2.73	2.22	+
Total people told per event	2.03	0.82	†	0.85	0.35	†
Secret keeping^a^						
Respondent kept secret (%)	7.36	31.01	†	12.71	24.68	†
If kept, mean number of people kept from	2.61	2.63		3.66	3.01	*
Total people secret kept from per event	0.20	0.82	†	0.47	0.74	†
N	278	179		1275	856	

*Note:* When male respondents were discussing their partner’s miscarriage or abortion, an additional person was added to who was told to account for the female partner telling the male respondent. If respondents indicated they had told someone but did not provide initials that would indicate how many people were told then they are treated as having told someone but are not contributing to how many people were told. Hence, the mean number are conservative.

a The secrets are kept from individuals with whom the respondent “usually talks about personal matters” per the survey question.

† *p* < 0.01;

* *p* < 0.05;

+ *p* < 0.10 (two tailed *t*-tests and tests of proportion were used to determine if there are significant differences between miscarriage and abortion).

**Table 2:** Relationship Patterns of Secret Telling and Secret Keeping of Own and Others’ Miscarriages and Abortions, AMACS 2012

	Respondent or Partner Has Had:		Diff.	Respondent Has Heard of Someone Else’s:		Diff.
	Miscarriage (%)	Abortion (%)		Miscarriage (%)	Abortion (%)	
Relationship to woman who experienced event						
Spouse				2.19	2.56	
Immediate family				19.26	11.06	^†^
Boyfriend or girlfriend				3.62	7.90	^†^
Other family				16.57	13.01	*
Close friend				14.14	15.86	
Other friend				16.86	16.68	
Acquaintance				27.35	32.92	^†^
Total^a^				99.99	99.99	
Source of information						
The woman				53.04	57.97	
The partner				7.86	5.12	
Someone else				39.11	36.38	
Total^a^				100.01	99.47	
Whom respondent told secret^b^						
Immediate family	93.91	73.65	^†^	86.57	79.95	*
Close friend	54.53	50.18		38.82	34.68	
Other	18.40	21.65		10.92	10.34	
Whom respondent kept secret from^b^						
Immediate family	81.36	82.30		54.55	70.40	*
Close friend	31.13	53.39		44.77	44.54	
Other	16.88	35.02		10.50	19.03	

a Due to rounding, totals may sum to more or less than 100.

b Respondents often told and avoided telling more than one person, hence the percentages for those parts of the table will sum to more than 100.

‡ *p* < 0.01;

* *p* < 0.05 (two tailed t-tests were used to determine significance between abortion and miscarriage).

**Table 3:** Odds Ratio for Reporting Knowing Someone Who Has Had an Abortion, AMACS 2012

	Model 1	Model 2	Model 3	Model 4
Abortion attitude (reference is generally available)				
Stricter limits	0.89 (0.16)	0.83 (0.16)	1.00 (0.21)	0.97 (0.20)
Rape/incest/life	0.59† (0.09)	0.53† (0.09)	0.66* (0.12)	0.63* (0.12)
Not at all	0.43† (0.09)	0.36† (0.08)	0.45† (0.12)	0.42† (0.12)
Number of miscarriage secrets heard	— —	1.50† (0.07)	— —	1.42† (0.08)
Controls	No	No	Yes	Yes
Constant	1.15 (0.22)	0.58* (0.13)	0.28* (0.17)	0.07† (0.05)
Observations	1607	1605	1496	1495

*Note:* (1) Standard errors are in parentheses. (2) Models 3 and 4 also control for age, gender, race, education, income, marital status, religion, religious service attendance, fundamentalist/evangelical, urban area, region, political party affiliation, gregariousness and randomization within the survey. (3) Model fit was diagnosed using Hosmer-Lemeshow’s *F* -adjusted mean residual test for logistic regression using sample survey data (Archer and Lemeshow 2006).

† *p* < 0.01;

* *p* < 0.05.

**Table 4:** Frequency of Why Secrets are Kept, AMACS 2012

	Respondent or Partner Has Had		Respondent Has Heard of Someone Else’s	
	Miscarriage (%)	Abortion (%)	Miscarriage (%)	Abortion (%)
Privacy	37.5	42.86	59.24	51.66
Asked to keep a secret			13.04†	28.91
Avoiding stigma	2.5†	36.13	2.01†	13.27
Number of people secret kept from	40	119	299	422

*Note:* Note: The responses can have more than one theme. Some responses are not included here due to not being important to the argument. Hence, the columns would sum to more than one hundred if all the themes were included but here they sum to less than 100.

† *p* < 0.01 (two tailed *t*-tests were used to determine significance between miscarriage and abortion).

## Footnotes

1 This inquiry examines two pieces of personal information, one of which is highly stigmatized, the second less so. Warren and Laslett (1977) would argue that the stigmatized information is a secret and the less stigmatized is a private matter. Readers may find it helpful to consider the distinction for this empirical inquiry, but I, like Nippert-Eng (2010), find it more conceptually helpful to consider secrets as a broader category of hidden information that includes information ranging in associated stigma.

2 Although secrets create gaps, not all gaps are the result of secrets. Some are the result of mere error. Intentionality differentiates the gap that results from a secret from the gap that results from an error.

3 Contact with a member of a stigmatized group is not the only way attitudes can shift. Coming into contact with someone who has a different attitude than one’s own can also shift attitudes (see the literature on interpersonal influence and attitudes or cross-cutting conversations in Katz and Lazarsfeld [1955], Huckfeldt and Sprague [1995], Huckfeldt, Johnson, and Sprague [2004], Huckfeldt, Ikeda, and Pappi [2005], Huckfeldt [2007], Mutz [2002a, 2002b, 2006], Sunstein [2009], and Baldassarri [2009]).

4 Scholars have outlined other optimal conditions, though these are not necessary for intergroup contact to change attitudes (Allport 1954; Williams 1947; Brewer and Miller 1984, 1988; Miller 2002; Brown and Hewstone 2005; Gaertner and Dovidio 2000; Gaertner et al. 1989, among many others).

5 There are, of course, other ways in which people do not know the full truth of others’ lives such as a result of mere lack of information (Baldassarri and Bearman 2007), bias, or error (Goel, Mason, and Watts 2010; Jussim and Osgood 1989; Laumann 1969; Eagly et al. 1999). Secrets differ meaningfully from these because they are deliberate attempts to create or preserve misperception of others.

6 The focus of this article is on who hears what secrets; as such, the discussion in this article will focus only on the aspects of secrets and secrecy relevant to communication. Hence, a number of elements of secrets and secrecy are outside the scope of this endeavor and will not be addressed, such as the social structure of those implicated in a secret (as a recent example, see Bruinsma and Bernasco 2004) and the choices those people make with regard to outside pressures (as a recent example, see Morselli, Giguère and Petit 2007), the revelation of secrets in data collection (Weinreb 2006), or the norms that make something worth concealing in the first place.

7 Definitions of gossip vary; some include discussions of people either present or absent (Dunbar, Marriott, and Duncan 1997), whereas others only consider discussion of absent third parties (Hannerz 1967; Eder and Enke 1991). Some definitions include all discussion, whereas others only negative evaluative discussion (Feinberg et al. 2012; Foster [2004] has an extensive discussion of varying definitions of gossip). The definition that includes only negative evaluative discussion of absent third parties is the closest to the layman’s use of the word gossip and is most often used. Gossip does not necessarily entail the disclosure of a secret; two friends discussing the extravagance of a third’s newest sports car constitutes gossip but not the revelation of a secret. Furthermore, sharing a secret does not necessarily entail negative evaluations; a friend may share another’s secret so they together can provide help, without judgment. Despite these reservations, the insights from the gossip literature can be instructive.

8 This question captures respondent attitudes toward abortion legality. Another question captures respondent attitudes toward abortion morality. The analyses presented here only address legality but were replicated with regard to morality and the trends remain the same.

9 For parsimony’s sake, the scale is collapsed to categories of “very gregarious,” “gregarious,” and “not gregarious” for the bivariate statistics.

10 Separate analyses were done on a sample of respondents who knew about the pregnancies in advance of the miscarriage or abortion and on a sample of respondents who did not know about the pregnancies in advance. The same patterns are found as in the sample as a whole, indicating these patterns are not due to disclosure of pregnancies.

11 I also tested for interactions between attitude toward abortion and randomization within the survey, and this does not have a substantive effect either.

12 The intimate relationship may be an example of notification norms as described in Ryan (2006).
